# ST2 blockade mitigates peritoneal fibrosis induced by TGF‐β and high glucose

**DOI:** 10.1111/jcmm.14571

**Published:** 2019-08-09

**Authors:** Yong Chul Kim, Kyu Hong Kim, Sunhwa Lee, Ji‐won Jo, Jae Yoon Park, Mi‐seon Park, Bodokhsuren Tsogbadrakh, Jung Pyo Lee, Jae Wook Lee, Dong Ki Kim, Kook‐Hwan Oh, In‐Jin Jang, Yon Su Kim, Ran‐hui Cha, Seung Hee Yang

**Affiliations:** ^1^ Department of Internal Medicine Seoul National University Hospital Seoul Korea; ^2^ Department of Microbiology The Ohio State University Columbus OH USA; ^3^ Biomedical Research Institute, Seoul National University Hospital Seoul Korea; ^4^ Department of Internal Medicine Dongguk University Ilsan Hospital Gyeonggi‐do Korea; ^5^ Department of Internal Medicine Seoul National University Boramae Medical Center Seoul Korea; ^6^ Department of Internal Medicine Seoul National University College of Medicine Seoul Korea; ^7^ Nephrology Clinic National Cancer Center Ilsan Korea; ^8^ Kidney Research Institute, Seoul National University Seoul Korea; ^9^ Department of Clinical Pharmacology and Therapeutics Seoul National University College of Medicine and Hospital Seoul Korea; ^10^ Department of Medical Science Seoul National University College of Medicine Seoul Korea; ^11^ Division of Nephrology, Department of Internal Medicine National Medical Center Seoul Korea

**Keywords:** peritoneal dialysis, peritoneal fibrosis, soluble ST2, ST2 blockade

## Abstract

Peritoneal fibrosis (PF) is an intractable complication of peritoneal dialysis (PD) that leads to peritoneal membrane failure. This study investigated the role of suppression of tumorigenicity (ST)2 in PF using patient samples along with mouse and cell‐based models. Baseline dialysate soluble (s)ST2 level in patients measured 1 month after PD initiation was 2063.4 ± 2457.8 pg/mL; patients who switched to haemodialysis had elevated sST2 levels in peritoneal effluent (1576.2 ± 199.9 pg/mL, *P* = .03), which was associated with PD failure (*P* = .04). Baseline sST2 showed good performance in predicting PD failure (area under the receiver operating characteristic curve = 0.780, *P* = .001). In mice with chlorhexidine gluconate‐induced PF, ST2 was expressed in fibroblasts and mesothelial cells within submesothelial zones. In primary cultured human peritoneal mesothelial cells (HPMCs), transforming growth factor‐β treatment increased ST2, fibronectin, β‐galactosidase and Snail protein levels and decreased E‐cadherin level. Anti‐ST2 antibody administration reversed the up‐regulation of ST2 and fibronectin expression; it also reduced fibrosis induced by high glucose (100 mmol/L) in HPMCs. Thus, high ST2 level in dialysate is a marker for fibrosis and inflammation during peritoneal injury, and blocking ST2 may be an effective therapeutic strategy for renal preservation.

## 1 INTRODUCTION

Peritoneal fibrosis (PF) is a major complication of peritoneal dialysis (PD) that undermines peritoneal membrane function, eventually necessitating the discontinuation of PD.^1^, ^2^, ^3^, ^4^, ^5^ Conventional glucose‐based PD solutions are inexpensive, safe and effective for osmotic fluid removal, but the high glucose (HG) concentrations, glucose degradation products and acidity may be harmful. Prolonged exposure of the peritoneal membrane to a bio‐incompatible dialysis solution and repeated episodes of peritonitis or haemoperitoneum can cause peritoneal injury resulting in PF.^2^, ^6^

Peritoneal fibrosis is characterized by fibroproliferative changes in the peritoneal membrane, the denudation and altered appearance of peritoneal mesothelial cells (PMCs), accumulation of extracellular matrix (ECM) molecules in submesothelial areas and vasculopathy.^7^, ^8^, ^9^ The key mediator of this process is transforming growth factor (TGF)‐β. Mesothelial cells synthesize ECM molecules including collagens I, III and IV, and fibronectin when exposed to TGF‐β.^10^ The TGF‐β signalling pathway is transduced via its downstream effectors SNAIL, p65, β‐galactosidase and connective tissue growth factor/cysteine‐rich protein 61/nephroblastoma‐overexpressed gene (CCN)1 to mediate fibrosis, inflammation and senescence.^11^ A high concentration of glucose was shown to directly activate TGF‐β and stimulate fibronectin synthesis in PMCs, leading to ECM accumulation and PF.^12^, ^13^

Suppression of tumorigenicity (ST)2 is an interleukin (IL)‐1 receptor family member that exists as soluble (sST2) and trans‐membrane (ST2L) isoforms. Recent studies have shown that the IL‐33/ST2 pathway plays an important role in inflammation and fibrosis in various organs such as heart,^14^ lung,^15^ gastrointestinal tract 16 and liver 17; moreover, sST2 is a useful biomarker for predicting clinical outcome in graft‐vs‐host disease (GVHD),^18^, ^19^ myocardial infarction 20 and heart failure 21 and kidney disorders such as IgA nephropathy,^22^ lupus nephritis 23 and chronic kidney disease.^24^, ^25^, ^26^ However, the role of ST2 in PF has not been previously reported.

To address this issue, the present study investigated the association between PD effluent levels of sST2 and PD failure (PDF) and the mechanism of action of ST2 in PF using in vitro and in vivo systems.

## 2 MATERIALS AND METHODS

### 2.1 Study population

The study protocol received full approval from the institutional review board of Seoul National University Hospital (no. H‐1701‐133‐829). All procedures were performed in accordance with the ethical standards of the institutional and/or national research committee and with the 1964 Declaration of Helsinki and its later amendments or comparable ethical standards. Informed consent was obtained from study participants, and blood and peritoneal effluent samples were collected, stored and monitored by the Seoul National University Hospital Human Biobank.

A total of 75 end‐stage renal disease (ESRD) patients with PD were enrolled between January 2010 and December 2016. Demographic and clinical information at the time of diagnosis including age, gender, body mass index (BMI) and comorbidities (eg hypertension and diabetes mellitus [DM]) was extracted from electronic medical records. Samples and clinical data were collected at four time‐points: baseline (defined as the first 6 months of PD) and at 1, 2 and 3 consecutive years. Exclusion criteria were age under 18 years, solid or haematological malignancies, and kidney transplantation within 3 months.

### 2.2 Biochemical parameters

Biochemical parameters including blood haemoglobin and serum albumin and creatinine (Cr) levels were measured using a Modular D2400 analyser with ISE900 module (Hitachi) and a cobas 8000 modular analyser (Roche Diagnostics).

### 2.3 Measurement of peritoneal effluent sST2 levels by enzyme‐linked immunosorbent assay

sSuppression of tumorigenicity (sST)2 levels were measured with an ELISA kit (R&D Systems; cat. no. M3300) according to the manufacturer's instructions. The lower limit of detection for sST2 was 31.3 pg/mL.

### 2.4 Estimation of peritoneal membrane function and definition of PDF

Peritoneal transport was estimated by calculating small solute transport (urea and Cr dialysate‐to‐plasma ratio [D/P]) under 4.25% glucose exchange with a 4‐hours dwell time, and weekly Kt/V ([dialyser clearance of urea × dialysis time]/volume of distribution of urea) was estimated by 24‐hour effluent dialysate and urine collection. Both tests were performed 1 month after the initiation of PD. Serum and effluent samples were stored at −80°C after each analysis of peritoneal kinetics. We defined PDF as a modality change to haemodialysis mainly due to ultrafiltration failure.

### 2.5 Isolation and primary culture of HPMCs

HPMCs from PD patients were obtained from PD effluent as previously described.^27^, ^28^ Briefly, effluent from clinically stable patients was drained and immediately processed. Bags were suspended for 3‐4 hours in an incubator at 37°C to allow the cells to settle at the bottom. The supernatant was removed by vacuum with a sterile pipette, leaving approximately 200 mL of sediment. The cells were transferred to four 50‐mL tubes, centrifuged at 1500 rpm for 20 minutes and washed twice with phosphate‐buffered saline (PBS). The cell pellets were resuspended in 5‐7 mL culture medium, counted in a Neubauer chamber, seeded in 25‐cm^2^ tissue culture flasks and incubated at 37°C in a humidified atmosphere with 5% CO_2_. The culture medium was M199 (Biological Industries) supplemented with 20% foetal bovine serum, 100 IU/mL penicillin, 100 mg/mL streptomycin and 2% Biogro‐2 (Biological Industries), which contains insulin, transferrin, ethanolamine and putrescine. The medium was replaced every 2‐3 days. Peritoneal leucocytes that adhered to the plates were detached after 48 hours and removed in subsequent washes.

HPMCs from the second passage were used for experiments. Primary cultured HPMCs were seeded in six‐well plates or 10‐cm^2^ plastic culture dishes in Dulbecco's modified Eagle's medium/F12 under normal glucose or HG conditions. When the cells reached 70%‐80% confluence, they were serum‐starved for 24 hours to synchronize cell growth.

### 2.6 Fibrosis induction and ST2 blocking antibody (Ab) administration

Peritoneal fibrosis was induced in primary cultured HPMCs with 1, 2 and 4 ng/mL recombinant (r)TGF‐β (R&D Systems) or by administration of low glucose (100 mmol/L) and HG (200 mmol/L) solutions for 72 or 120 hours. The cells were simultaneously treated with anti‐ST2 monoclonal (m)Ab (Janssen Biotech; CNT03914) at 0.5 or 1.0 mg/mL.

### 2.7 Western blot analysis

HPMCs were harvested from culture dishes, and proteins were extracted using radioimmunoprecipitation assay buffer containing Halt protease inhibitor (Pierce). Immunoblotting was performed with primary antibodies against fibronectin, ST2 and β‐galactosidase (all from Santa Cruz Biotechnology); E‐cadherin, Snail, collagen 4, alpha‐smooth muscle actin (αSMA), p65, phosphorylated (p‐)p65, CCN1, GAPDH and IL‐33 (all from Abcam); and β‐actin (Sigma‐Aldrich). Equal amounts (30 μg) of extracted protein were separated by 10% sodium dodecyl sulphate‐polyacrylamide gel electrophoresis and transferred to an Immobilon‐FL 0.4‐μm polyvinylidene difluoride membrane (Millipore). Horseradish peroxidase‐conjugated anti‐rabbit and antimouse IgG (both from Cell Signaling Technology) were used as secondary antibodies. Labelled proteins were detected by enhanced chemiluminescence (Amersham Pharmacia Biotech; ECLTM PRN 2106) using a Gel Doc 1000 imager with Multi‐Analyst v.1.1 software (Bio‐Rad).

### 2.8 Fluorescence‐activated cell sorting (FACS)

Primary cultured HPMCs were sorted using a FACSCalibur instrument (BD Biosciences). Cells were cultured in Ham's F12 medium supplemented with 10% foetal bovine serum (Gibco), 1% penicillin/streptomycin, 2% HEPES and endothelial cell growth medium (Lonza; CC‐3124). For the experiment, HPMCs (5 × 10^5^) were added to a 1.5‐mL tube, and the culture medium was removed. A 4 µL volume of anti‐ST2 or αSMA mAb (Abcam) was diluted in 100 µL PBS, and the cell suspension was incubated in this solution at 4°C for 30 minutes. The cells were centrifuged at 4000 rpm for 3 minutes and washed twice, and 2 µL of phycoerythrin‐conjugated anti‐human IgG (Southern Biotech) was added to the suspension. The cells were washed and resuspended in 1 mL PBS and subjected to FACS analysis. Data were acquired with the BD FACSCanto platform (BD Biosciences) and analysed with FlowJo 10.0.7 (FlowJo LLC) and BD FACSDiva v.8.0 (BD Biosciences) software programs.

### 2.9 Animals and treatments

Animal experiments were performed with the approval of the Institutional Animal Care and Use Committee (IACUC) of Seoul National University Hospital (approval no. 12‐0094). Male C57BL/6 mice (The Jackson Laboratory) weighing 20 g were divided into two groups (n = 6/group). PF was induced by daily intraperitoneal injection of 0.2 mL chlorhexidine solution composed of 0.1% chlorhexidine gluconate (CG) and 15% ethanol for 4 weeks (16). Mice were maintained in compliance with IACUC protocols. In week four, mice were anaesthetized and the peritoneal tissue was dissected. The peritoneum at the pole was embedded in paraffin for immunostaining or snap‐frozen in liquid nitrogen for immunoblotting. B6.ST2 KO mice were provided by Andrew McKenzie from University of Cambridge, UK.

### 2.10 Real‐time quantitative PCR analysis

Total RNA was extracted from the peritoneum, and the mRNA levels of target genes were assayed by real‐time quantitative PCR. Briefly, total RNA was isolated from the peritoneum using the RNeasy kit (Qiagen GmBH), and 500 ng of total RNA was reverse‐transcribed using oligo‐d(T) primers and AMV‐RT Taq polymerase (Promega). Real‐time qPCR was conducted on an ABI PRISM 7500 sequence detection system using either Assay‐on‐Demand TaqMan probes and primers (fibronectin, ST2) or the SYBR Green method (for GAPDH, fibronectin, periostin, TGF‐β, NF‐κB, Nrf‐2, MCP‐1, collagen 1, Bax, BCL2, P53 primer sequences are available in Table S1) (Applied Biosystems). Relative quantification was performed with the 2^‐∆∆CT^ method. GAPDH was used as a loading control. All experiments were completed in triplicate.

### 2.11 Statistical analysis

Results are expressed as mean ± SD or SEM. Statistical analyses were performed with Prism v.8.0 software (Graph Pad Inc.) and SPSS for Windows v.25.0 (SPSS Inc.).

The patients were classified into two groups according to a peritoneal effluent sST2 cut‐off of 1155 pg/mL, which was calculated from receiver operating characteristic (ROC) curves. Categorical variables expressed as frequencies and proportions were compared with the chi‐squared test. Normally distributed continuous variables are expressed as mean ± SD and were compared with the Student's *t* test or by one‐way analysis of variance. Non‐normally distributed variables are expressed as median (25th and 75th percentiles) and were compared with the Mann‐Whitney U or Kruskal‐Wallis test. A *P* value < .05 was considered statistically significant.

To evaluate the impact of peritoneal effluent sST2 levels on PDF, Cox proportional hazard models with time‐fixed peritoneal effluent sST2 levels at the time of PD initiation were used for multivariate survival analyses. Significant covariates identified in the univariate analysis and clinically important covariates were included in the final multivariable‐adjusted analysis, which was carried out in a backward stepwise manner. The adjusted covariates were age, sex, BMI, DM, hypertension, haemoglobin, serum albumin, Cr, baseline weekly total Kt/V and baseline D/P Cr

## 3 RESULTS

### 3.1 Patients who develop PDF show higher dialysate levels of sST2

Patient baseline characteristics are shown in Table 1. A total of 75 patients were enrolled in this study. During the 59.4 ± 16.8 months of follow‐up, 10 patients switched to haemodialysis due to PDF. Diabetes mellitus was the most frequent cause of ESRD in patients who developed PDF. Other baseline variables including weekly Kt/V and D/P Cr showed no differences among groups.

**Table 1:** Baseline characteristics of PD patients

Variable	Total (n = 75)	No PDF (n = 65)	PDF (n = 10)	*P* value
Follow‐up duration (mo)	59.4 ± 16.8	58.3 ± 17.0	67.1 ± 13.4	NS
Age (y)	51.2 ± 14.7	50.5 ± 14.7	55.5 ± 14.8	NS
Sex (male, %)	49 (65.3)	45 (69.2)	4 (40)	NS
BMI (kg/m^2^)	22.5 ± 3.2	22.5 ± 3.3	23.0 ± 2.2	NS
Diabetes, n (%)	14 (21.5)	14 (21.5)	5 (50)	NS
Hypertension, n (%)	59 (90.8)	59 (90.8)	9 (90)	NS
Cause of ESRD, n (%)				.030
Diabetes	16 (21.3)	11 (16.9)	5 (50.0)	
Hypertension	13 (17.3)	12 (18.5)	1 (10.0)	
Glomerulonephritis	32 (42.7)	28 (43.1)	4 (40.0)	
Other	14 (18.7)	14 (21.5)	0 (0.0)	
Blood haemoglobin (g/dL)	9.82 ± 1.68	9.82 ± 1.64	9.66 ± 2.01	NS
Serum albumin (g/dL)	3.67 ± 0.55	3.70 ± 0.55	3.47 ± 0.54	NS
Creatinine (mg/dL)	8.89 ± 4.93	9.00 ± 5.19	8.10 ± 2.52	NS
hs‐CRP (mg/dL)	0.40 ± 0.78	0.38 ± 0.75	0.75 ± 1.06	NS
Weekly Kt/V (PD)	1.18 ± 0.43	1.15 ± 0.40	1.34 ± 0.56	NS
Weekly Kt/V (Renal)	0.96 ± 0.56	0.95 ± 0.55	1.00 ± 0.65	NS
Weekly Kt/V (Total)	2.13 ± 0.56	2.10 ± 0.58	2.33 ± 0.44	NS
D/P creatinine	0.71 ± 0.16	0.70 ± 0.16	0.73 ± 0.18	NS
D/P urea	0.93 ± 0.11	0.93 ± 0.11	0.96 ± 0.16	NS
Dialysate sST2 (pg/mL)	2063.4 ± 2457.8	1663.1 ± 1571.0	4665.5 ± 4841.4	<.001

Note

Values represent mean ± SD or number (percentage) unless otherwise indicated.

Abbreviations: BMI, body mass index; D/P, dialysate‐to‐plasma ratio; ESRD, end‐stage renal disease; hs‐CRP, high‐sensitivity C‐reactive protein; Kt/V, (dialyser clearance of urea × dialysis time)/volume of distribution of urea; NS; not significant; PDF, peritoneal dialysis failure.

**Figure 1:**
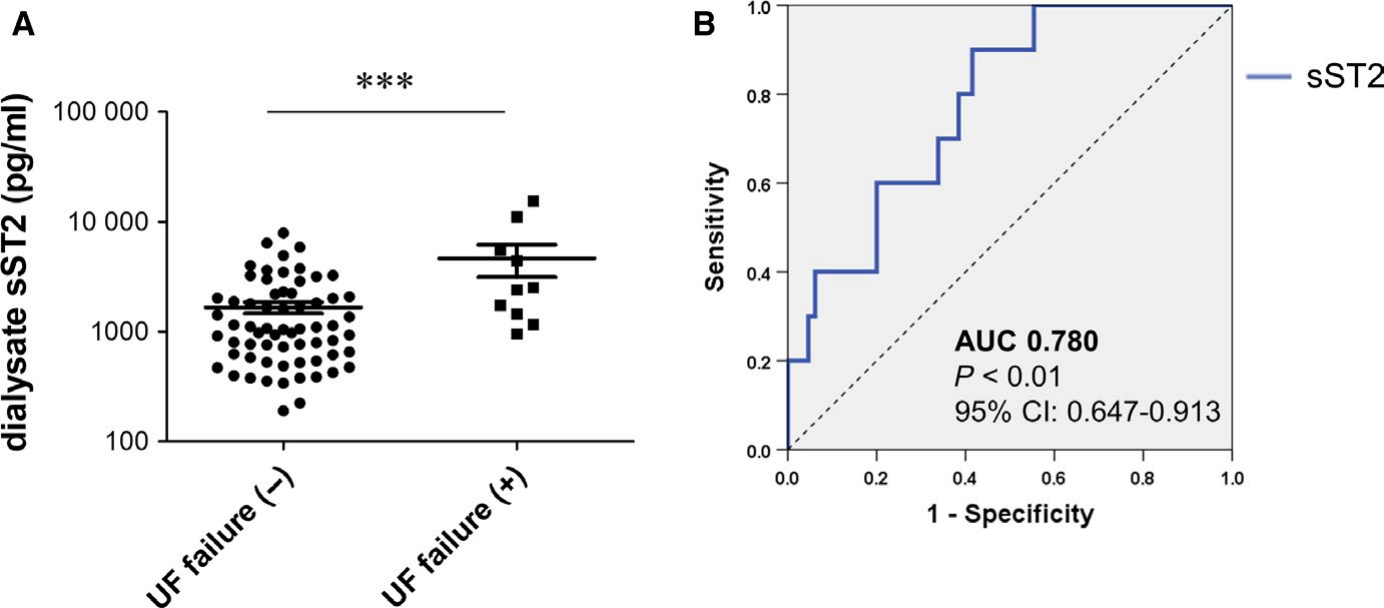
Effluent sST2 concentrations predict survival of PD patients. A, High baseline sST2 levels were detected in peritoneal effluent samples from patients who changed their dialysis modality to haemodialysis due to PDF (n = 10) as compared to other patients (n = 65). B, ROC curve analysis of baseline sST2 effluent concentrations in samples from patients at the start of PD treatment who did or did not develop PDF. AUC, area under the curve; CI, confidence interval

Baseline dialysate sST2 concentration was higher in the 10 patients who eventually developed PDF (no PDF: 1663.1 ± 1571.0 pg/mL vs. PDF: 4665.5 ± 4841.4 pg/mL; *P* < .001). An ROC curve was generated to assess the capacity of baseline sST2 in effluent to identify patients at risk of PDF. The area under the curve was 0.780 (95% confidence interval: 0.647‐0.913, *P* < .01). The optimal baseline sST2 value in effluent that simultaneously maximized sensitivity (90%) and specificity (58.5%) to predict subsequent development of PDF was 1155 pg/mL or higher (Figure 1). A multivariate analysis revealed that baseline sST2 level in effluent was independently associated with risk of PDF development after adjusting for peritoneal transport parameters (weekly Kt/V and D/P Cr) and clinical covariates such as age, sex, BMI, DM, hypertension, haemoglobin, serum albumin and Cr (Table 2).

**Table 2:** Association between peritoneal effluent sST2 level and peritoneal dialysis failure based on a Cox proportional hazard model

Variable	Hazard ratio (95% confidence interval)^a^	*P* value
Dialysate sST2^b^	8.87 (1.08, 72.25)	.04
Age	1.00 (0.93, 1.08)	NS
Sex	3.37 (0.85, 13.36)	NS
Diabetes mellitus	3.18 (0.66, 15.45)	NS
Weekly Kt/V (total)	1.69 (0.60, 4.80)	NS

Abbreviations: Kt/V, (dialyser clearance of urea × dialysis time)/volume of distribution of urea; sST2, suppression of tumorigenicity.

a Multivariate cox proportional hazard ratios adjusted for age, sex, body mass index, DM, hypertension, haemoglobin, serum albumin, creatinine, baseline weekly total Kt/V, baseline dialysate‐to‐plasma ratio of creatinine.

b Analyses were performed by including the predefined cut‐off value of peritoneal effluent sST2 according to the receiver operating characteristic curve; the cut‐off value was 1155 pg/mL with regard to peritoneal dialysis failure.

### 3.2 TGF‐β induces fibrosis in primary cultured HPMCs

**Figure 2:**
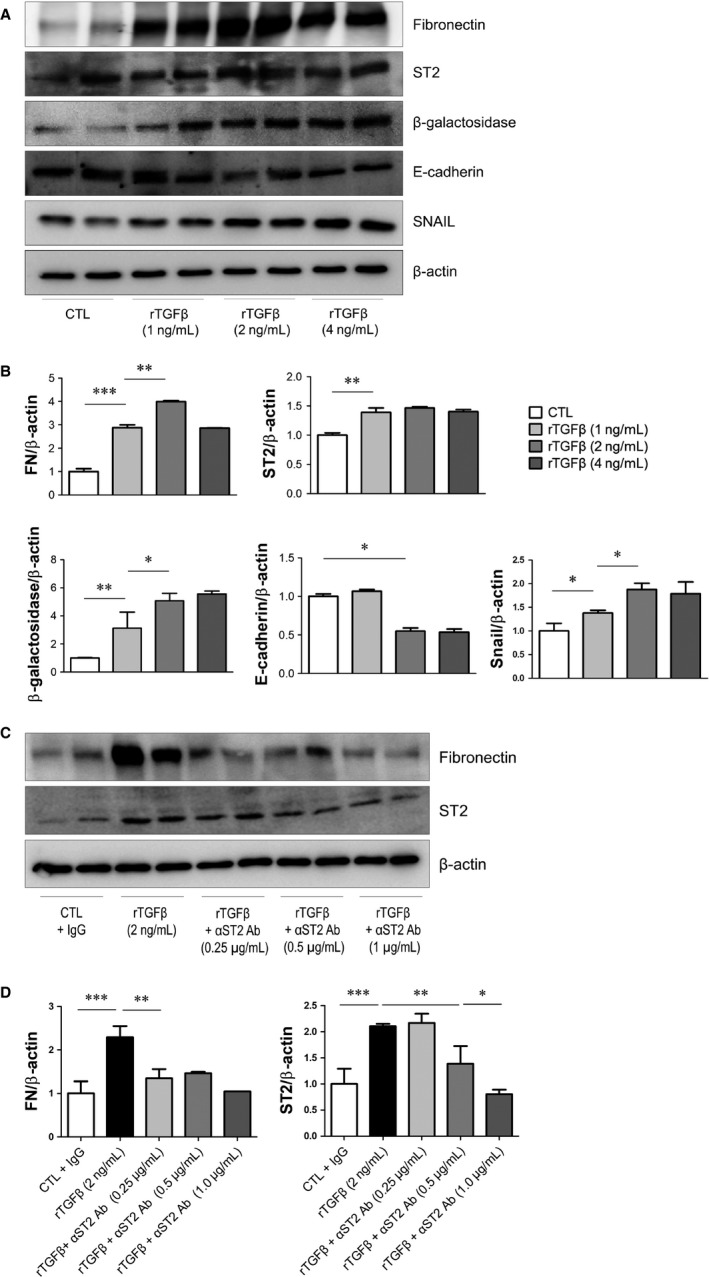
Effect of ST2 blockade induced by recombinant TGF‐β in HPMCs. (A, B) Western blot analysis of primary cultured HPMCs after induction of fibrosis with various concentrations of rTGF‐β (1, 2 and 4 ng/mL). rTGF‐β increased fibronectin, β‐galactosidase and Snail and decreased E‐cadherin protein expression. (C, D) Pre‐treatment with ST2 mAb (0.25, 0.5 and 1.0 μg/mL) for 30 min mitigated the rTGF‐β–induced increase in fibronectin and ST2 protein levels in a dose‐dependent manner. Data represent mean ± SD of three independent experiments. *P < .05, **P < .01 and ***P < .001 vs control group

To confirm that ST2 is expressed during PF in HPMCs, the cells were treated with rTGF‐β (1, 2 and 4 ng/mL) and ST2 protein expression was evaluated by Western blotting. ST2 was up‐regulated by rTGF‐β treatment; this was accompanied by increases in fibronectin, β‐galactosidase and Snail and a decrease in E‐cadherin expression (Figure 2A,B). These results indicate that TGF‐β induces fibrosis in HPMCs, with up‐regulation of ST2.

### 3.3 ST2 blockade attenuates TGF‐β–induced epithelial‐to‐mesenchymal transition (EMT) in HPMCs

To evaluate the role of ST2 in fibrosis, rTGF‐β–stimulated HPMCs were pre‐treated with ST2 blocking Ab. As expected, ST2 blockade suppressed the rTGF‐β–induced increase in sST2 expression in a dose‐dependent manner, with a concomitant decrease in fibronectin level (Figure 2C,D). Thus, TGF‐β induces EMT in HPMCs, an effect that is abrogated by blocking ST2 function.

### 3.4 HG concentration induces fibrosis in primary cultured HPMCs

**Figure 3:**
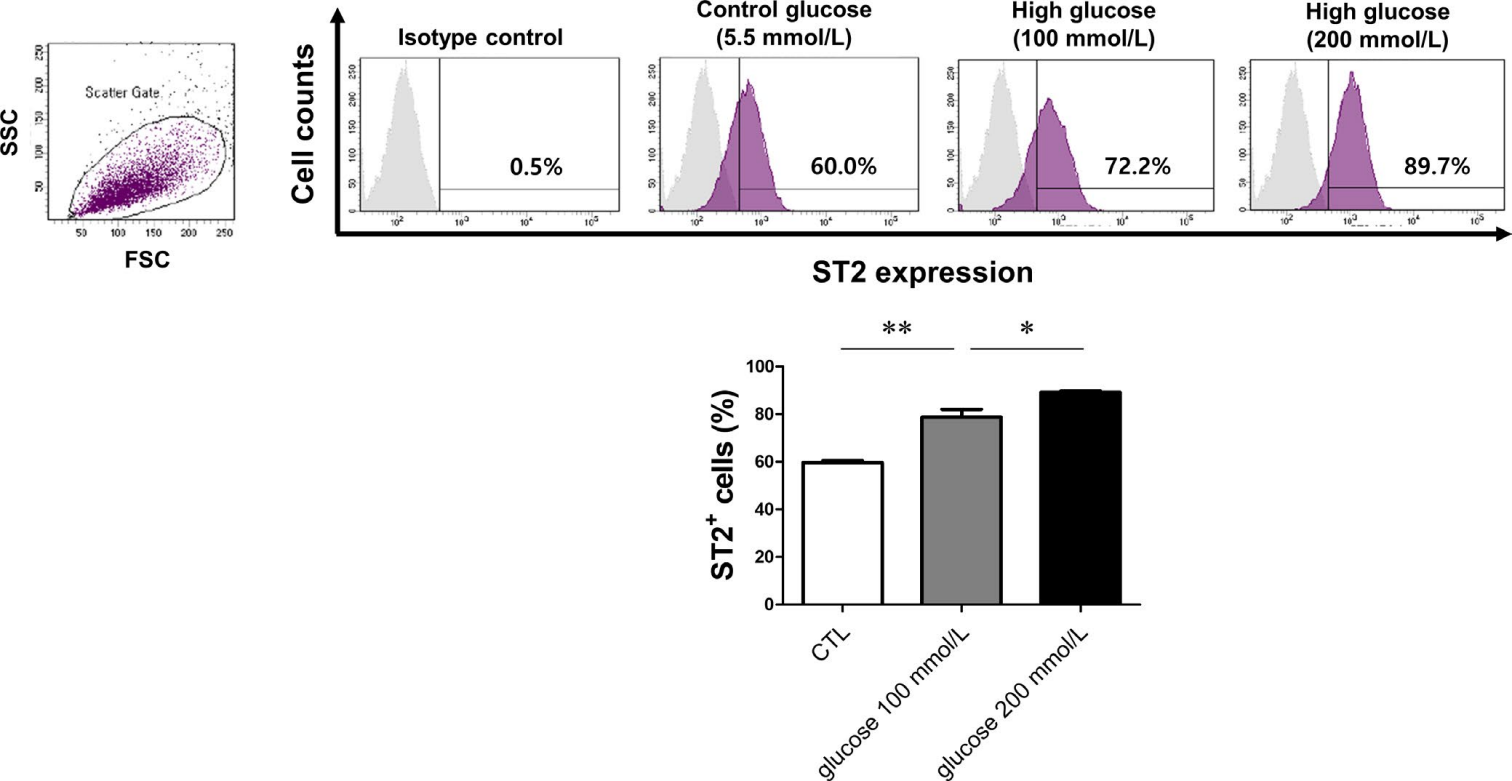
HG solution induces ST2 expression in HPMCs. Cells were treated with normal glucose (5.5 mmol/L) and HG (100 and 200 mmol/L) solutions for 72 h. The number of ST2‐positive cells was increased in a dose‐dependent manner by HG treatment, as determined by flow cytometry. Data represent mean ± SD of three independent experiments. * *P* < .05 and **P < .01 vs control group

**Figure 4:**
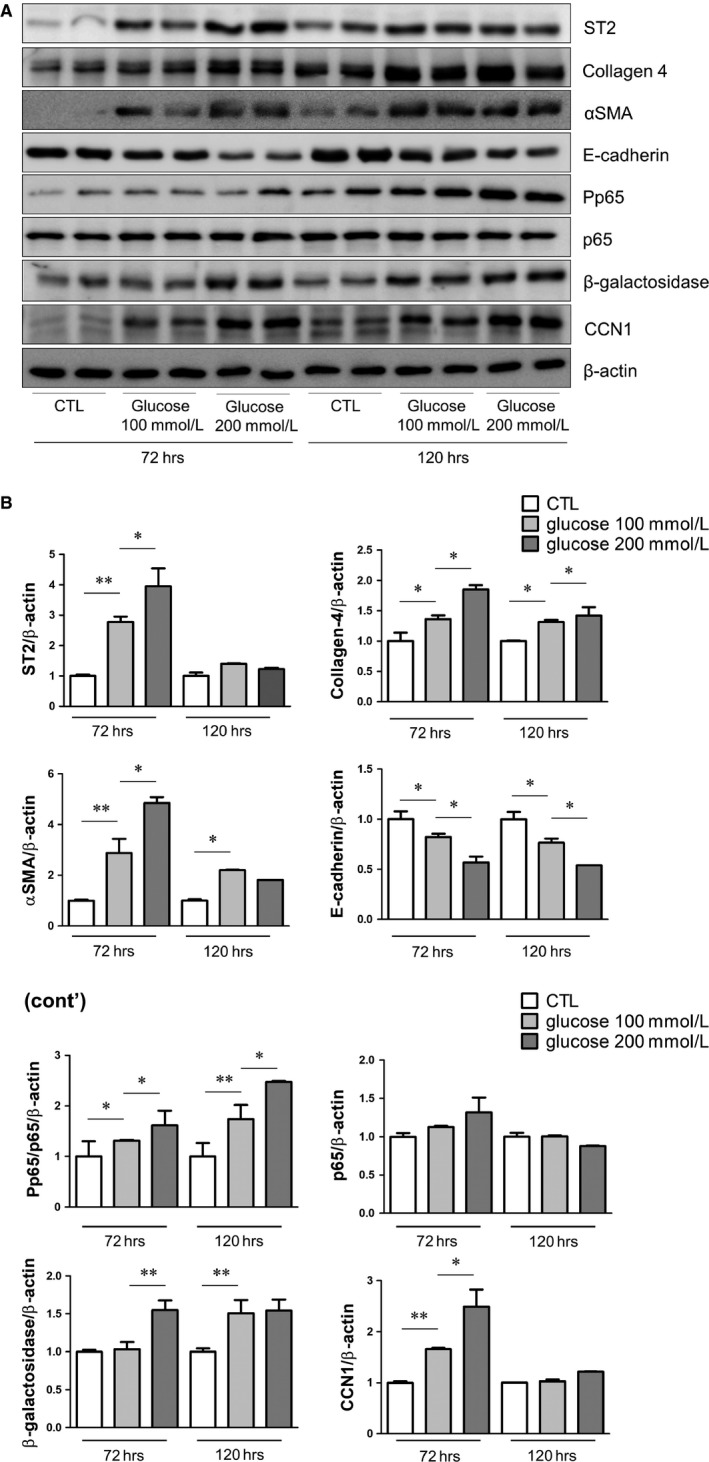
HG solution induces fibrosis, apoptosis and inflammation in HPMCs. (A, B) HPMCs were treated with normal glucose (5.5 mmol/L) and HG (100 and 200 mmol/L) solutions for 72 and 120 h. High Glucose treatment induced the expression of ST2 and markers of fibrosis (collagen 4, αSMA and CCN1), inflammation (p65) and senescence (β‐galactosidase), as determined by Western blotting. Data represent mean ± SD of three independent experiments. *P < .05 and **P < .01 vs control group

We investigated whether ST2 plays a similar role in HG‐stimulated HPMCs by FACS analysis. The number of ST2‐positive HPMCs was increased upon treatment with HG solution (100 and 200 mmol/L), which is similar to commercial 2.5% and 4.25% PD solutions (Figure 3). High glucose stimulation of HPMCs for various times increased ST2, collagen 4, αSMA, p‐p65 β‐galactosidase and CCN1 protein expression, especially in groups treated for 72 hours (Figure 4A,B).

### 3.5 ST2 blockade reverses PF in HPMCs cultured under HG conditions

**Figure 5:**
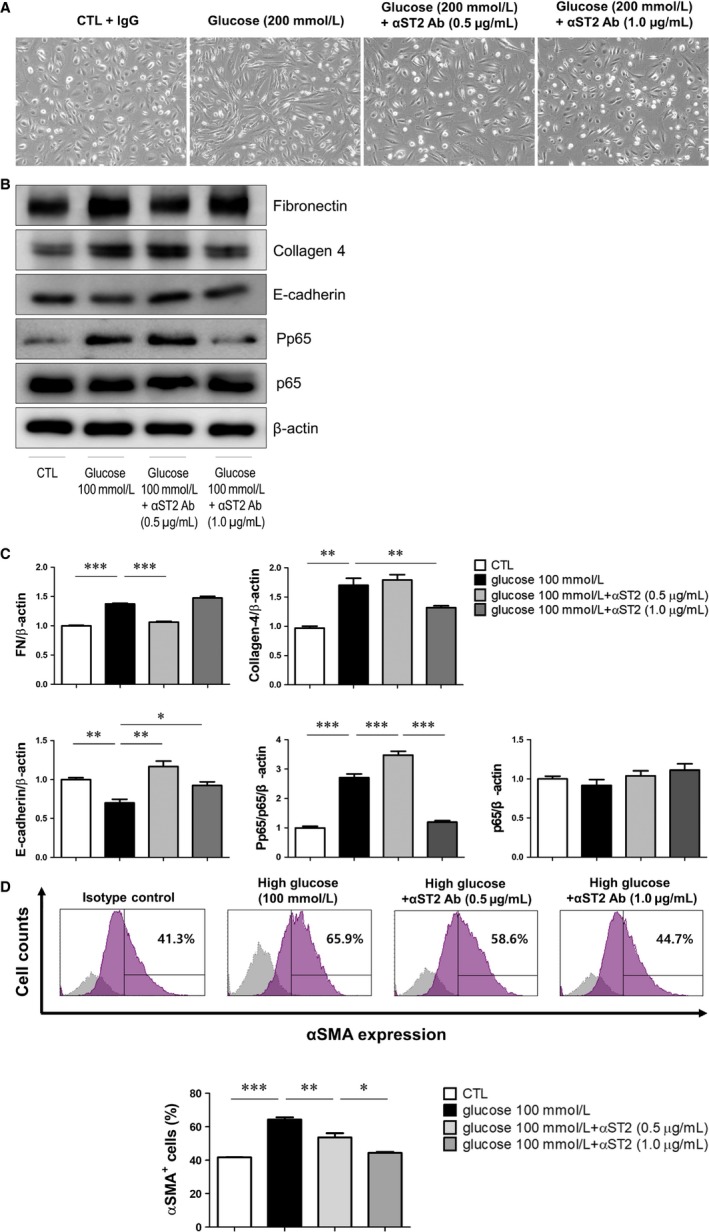
ST2 blockade reduces fibrosis in HPMCs. A, Morphological changes in primary cultured HPMCs treated with normal glucose (5.5 mmol/L) or HG (200 mmol/L) solutions for 72 h with or without pre‐treatment with anti‐ST2 blocking Ab (magnification: 100×). HG solution caused fibrotic changes that were alleviated by ST2 blockade. (B, C) HPMCs were exposed to 5.5 or 100 mmol/L glucose solutions for 72 h with or without anti‐ST2 blocking Ab pre‐treatment (0.5 and 1.0 µg/mL) for 30 min. The increase in fibronectin and collagen 4 and decrease in E‐cadherin expression induced by HG were reversed by ST2 blocking Ab treatment, as determined by Western blotting. D, HG solution increased the number of αSMA‐positive HPMCs, as detected by flow cytometry; this effect was abolished by ST2 blockade. Data represent mean ± SD of three independent experiments. *P < .05, **P < .01 and ***P < .001 vs control group

To determine whether ST2 blockade affects PF, we investigated changes in cell morphology and expression of fibrosis‐related molecules in HG‐stimulated HPMCs following treatment with ST2 mAb. HG (200 mmol/L) induced a fibroblast‐like morphology characterized by a spindle‐like shape, in contrast to the cuboidal form of unstimulated HPMCs (Figure 5A). Interestingly, administration of sST2 mAb (0.5 and 1.0 μg/mL) restored a normal cell morphology. Consistent with these results, sST2 mAb abrogated the up‐regulation of the mesenchymal markers fibronectin, collagen 4 and p‐p65 and down‐regulation of the epithelial marker E‐cadherin induced by HG (Figure 5B,C). Moreover, FACS analysis revealed that the proportion of αSMA‐positive HPMCs—which was increased under HG conditions—was reduced by ST2 blockade (Figure 5D). These results suggest that ST2 mediates HG‐induced fibrosis in HPMCs, which can be mitigated by ST2 inhibition.

### 3.6 ST2 expression in a mouse model of PF

**Figure 6:**
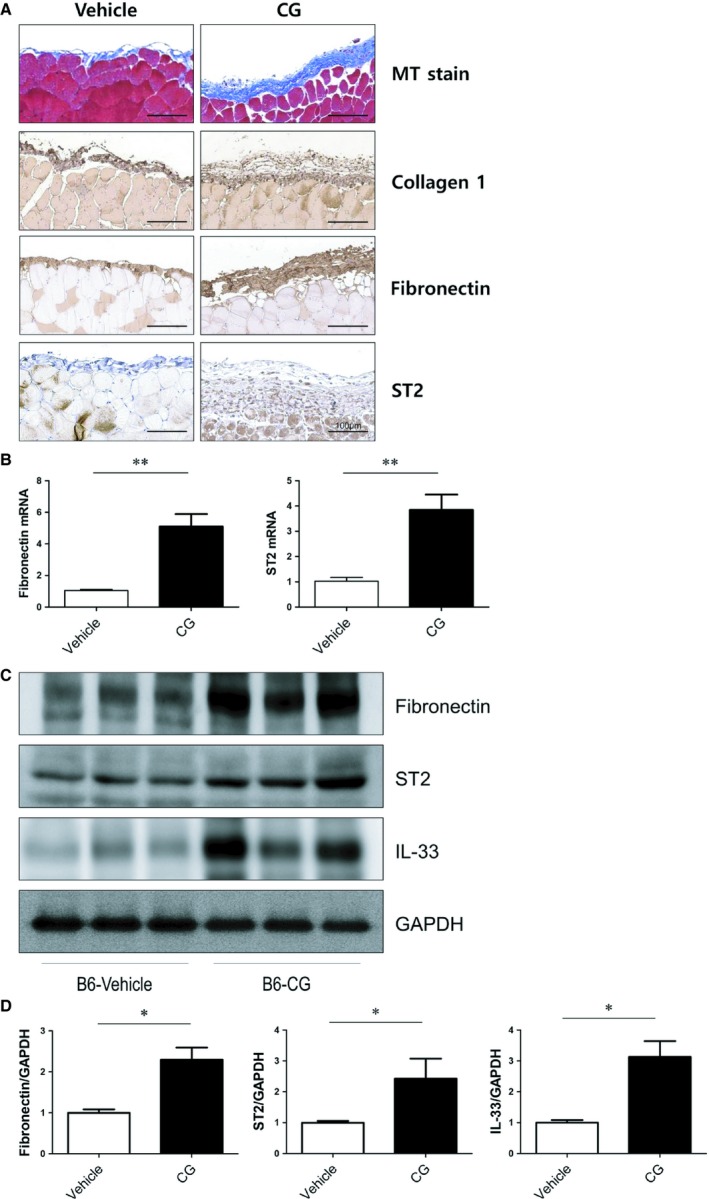
ST2 expression in a mouse model of PF. A, Examination of PF model mice by microscopy. C57BL/6J wild‐type mice (WT) treated with PBS showed no fibrosis in the peritoneum, whereas CG‐treated mice showed marked PF with moderate infiltration of mononuclear cells on day 14. Masson's trichome staining (blue) and immunohistochemical detection of collagen 1 and fibronectin (brown) revealed an increase in peritoneum thickness accompanying the up‐regulation of ST2 protein in the submesothelial layer in PF model mice (original magnification: 200×; scale bar: 100 μm). B, The mRNA expression of fibronectin and ST2 was significantly higher in the peritoneum of CG‐treated mice. (C, D) The protein expression of fibronectin, ST2 and IL‐33 was also increased in the peritoneum of PF model mice. *P < .05 and **P < .01 vs control group

To establish the expression profile of ST2 during PF progression, we carried out an immunohistochemical analysis of a mouse model of CG‐induced PF. In control mice, peritoneal tissues showed no thickening of the submesothelial area. However, CG‐treated mice showed elevated levels of collagen 1, fibronectin and ST2 in IHC staining (Figure 6A). Moreover, the mRNA expression of fibronectin and ST2 was also increased in the peritoneal tissue of CG‐treated mice (Figure 6B). In Western blot analysis, the protein expression of fibronectin, ST2 and IL‐33 was also significantly increased, which is in agreement with our in vitro findings (Figure 6C,D).

### 3.7 Amelioration of PF in ST2 knockout mice

**Figure 7:**
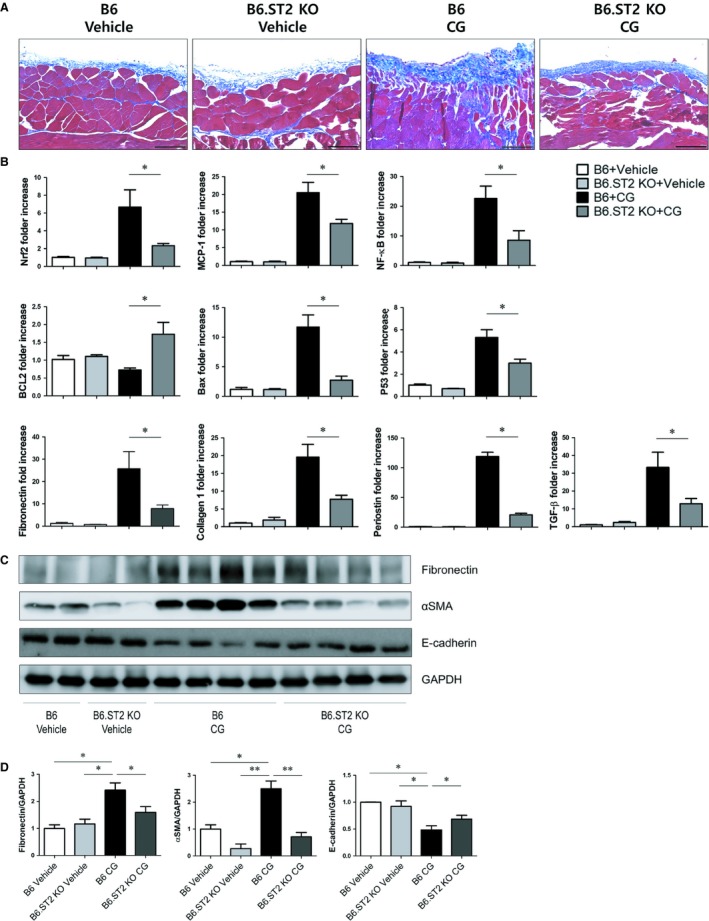
Deletion of ST2 showed anti‐fibrotic effect in PF mice. A, Representative microscopic images of the peritoneum at day 14 after induction of peritoneal fibrosis with CG (original magnification: 200×; scale bar: 100 μm). B, Peritoneal mRNA expression of Nrf‐2, MCP‐1, NF‐κB, Bax, P53, fibronectin, collagen 1, periostin and TGF‐β is increased, and BCL2 mRNA expression is decreased in PF mice model. ST2 deletion reverses mRNA expression patterns. (C, D) Increased fibronectin expression and αSMA protein expression induced by CG‐injection were attenuated by ST2 knockout. *P < .05 and **P < .01 vs control group

We finally examined whether deletion of ST2 could ameliorate PF in CG‐treated mice. There was no histological difference in the peritoneum between vehicle‐treated B6 mice and vehicle‐treated B6.ST2 KO mice. The peritoneum of CG‐treated B6 mice showed marked thickening and significant fibrosis on MT staining. In contrast, CG‐treated B6.ST2 KO mice revealed improvement of peritoneal thickening (Figure 7A). The mRNA expression of Nrf2, MCP‐1 and NF‐κB, which are known as pro‐inflammatory transcription factors, is decreased in ST2 KO mice. mRNA expression, which is related to apoptotic pathways, is expressed towards anti‐apoptotic patterns in ST2 KO mice. Furthermore, the mRNA expression of fibronectin, collagen 1, periostin and TGF‐β is decreased after ST2 deletion (Figure 7B). Chlorhexidine gluconate treatment induced protein expression of fibronectin and αSMA in B6 mice, and it was reduced in CG‐treated B6.ST2 KO mice (Figure 7C,D). These results suggest that the deletion of ST2 ameliorates peritoneal fibrosis not only by blocking anti‐fibrotic pathways, but also by modulating anti‐inflammatory and anti‐apoptotic signals.

## 4 DISCUSSION

The results of our study demonstrate for the first time the prognostic value of baseline dialysate sST2 levels in predicting PDF. Higher effluent sST2 level at 1 month after initiating PD was associated with increased risk of PDF after adjusting for comorbidities and demographic factors including baseline peritoneal membrane function parameters such as weekly total Kt/V and D/P Cr Consistent with these findings, ST2 was highly expressed in peritoneal tissue in a mouse model of PF. Inhibition of IL‐33/ST2 signalling by functional blockade of ST2 using an anti‐ST2 Ab abrogated rTGF‐β–induced PF in primary cultured HPMCs.

Soluble ST2 is a member of the IL‐1 receptor family,^29^ with IL‐33 as its only known ligand.^30^ sST2 level was found to be a useful biomarker of non‐response to GVHD therapy and improved risk stratification according to clinical grade.^31^ Meanwhile, baseline serum sST2 had good predictive value for heart failure 20 and myocardial infarction 21 of all subtypes and cardiovascular death in a large community‐dwelling population. These findings support our finding that elevated baseline sST2 level is associated with poor prognosis in ESRD patients who experience PDF.

During PD, peritoneal mesothelial cells are subjected to various insults including bio‐incompatible solutions, peritonitis, uraemia and chronic inflammation that can cause fibrotic changes in the peritoneum such as decreased efficiency of solute diffusion and dissipation of the osmotic gradient, ultimately leading to ultrafiltration failure. This significantly limits the widespread use of PD for long‐term renal replacement.^7^, ^32^, ^33^, ^34^

The most important finding of this study is the validation of the anti‐fibrotic effect of ST2 blockade in PF. Chronic inflammation is a key contributor to the pathogenic changes in peritoneal function. TGF‐β is a key factor in peritoneal inflammation and PF.^10^ It was previously thought that resident peritoneal fibroblasts and infiltrating inflammatory cells are the major mediators of PF. However, recent studies have shown that EMT of mesothelial cells is critical for the induction of fibrosis and subsequent deterioration of peritoneal function. Mesothelial cells undergo EMT into fibroblasts following repeated exposure to specific growth factors such as TGF‐β.^35^ EMT is one of the earliest events in the progression of PF; it begins with the breakdown of intercellular junctions due to down‐regulation of adhesion molecules such as E‐cadherin. Cells then adopt a front‐back polarity as a result of cytoskeletal reorganization and acquire αSMA expression. E‐cadherin gene expression is inhibited by Snail repressor, which is regulated by growth factors such as TGF‐β.^36^ In the present study, treatment of cultured HPMCs with rTGF‐β reduced the level of E‐cadherin and increased that of αSMA and Snail, which was accompanied by enhancement of fibronectin expression. ST2 blockade suppressed TGF‐β–induced changes in HPMCs by positively regulating E‐cadherin expression and negatively regulating that of αSMA and fibronectin. Thus, inhibiting EMT by blocking ST2 can prevent PF induced by TGF‐β and HG.

In summary, the results of this study demonstrate that high baseline sST2 level in PD effluent is associated with increased risk of PDF and that ST2 blockade can ameliorate PF. Thus, effluent sST2 level was found to be a promising biomarker for peritoneal damage, and therapeutic strategies that inhibit ST2 function may be effective in preventing PF development and progression.

## CONFLICT OF INTEREST

No conflicts of interest, financial or otherwise, are declared by the authors.

## AUTHORS' CONTRIBUTIONS

RC and SHY designed study. YCK, MP, BT, DKK and KO collected clinical samples. YCK, SL and MY analysed and interpreted the data. YCK, KHK, JJ and SHY performed the experiments. YCK, SL, MY and SHY wrote the manuscript. HL, JPL, DKK, KO, YSK, RC and SHY reviewed and revised the manuscript.
