# Supplementary material for: ST2 blockade mitigates peritoneal fibrosis induced by TGF‐β and high glucose
Source: J Cell Mol Med. 2019 Aug 9;23(10):6872–84. doi: 10.1111/jcmm.14571 (PMC6787438; doi:10.1111/jcmm.14571)
Supplement: Supplementary file 1 [file JCMM-23-6872-s001.pdf]

Supplementary Table 1. Sequences of real time PCR primers.

Target names	Species	Sense (5’-3’)	Antisense
GAPDH	MS	5’-TATGTCGTGGAGTCTACTGGT-3’	5’-GAGTTGTCATATTTCTCGT-3’
Fibronectin	MS	5’- TCCTGTCTACCTCACAGACTAC -3’	5’-GTCTACTCCACCGAACAACAA-3’
Periostin	MS	5′-TCCAGCAGATATTCCAGTTG-3′	5′-TTTCGCCTTCTTTAATCAGC-3′
TGFβ	MS	5’-ATGGTGAGCAATGGTATAGTG-3’	5’-GGGTCTGAGAACCATCTGTTAGG-3’
NF-κB	MS	5’-GGAATGTTCGGTAGTGG-3’	5’-CCCTGCGTTGGATTTCGTG-3’
Nrf-2	MS	5’-CTCGCTGGAAAAAGAAGTG-3’	5’-CCGTCCAGGAGTTCAGAGG-3’
MCP-1	MS	5’-GTGCTCCTGGTATTGCTGGT-3’	5’-GGCTCCTCGTTTTCCTTCTT-3’
collagen1	MS	5’-CCTGGTAAAGATGGTGCC-3’	5’-CACCAGGTTCACCTTTCGCACC-3’
Bax	MS	5’-CCCGAGAGGTCTTTTTCC-3’	5’-GCCTTGAGCACCAGTTTG-3’
BCL2	MS	5’-GGAAGGTAGTGTGTGTGG-3’	5’-ACTCCACTCTCTGGGTTCTTGG-3’
P53	MS	5’-GCCCATGCTACAGAGGAGTC-3’	5’- TGGTGATGTGGGACGGGAT-3’
